# Optimization and Impact of Ultrasound-Assisted Extraction on Pomegranate Seed Oil Quality: A Comparative Study of Bioactive Potential and Oxidation Parameters

**DOI:** 10.3390/molecules30081837

**Published:** 2025-04-19

**Authors:** Marta Siol, Iga Piasecka, Diana Mańko-Jurkowska, Agata Górska, Joanna Bryś

**Affiliations:** Department of Chemistry, Institute of Food Sciences, Warsaw University of Life Sciences, Nowoursynowska St. 159c, 02-787 Warsaw, Poland; marta_siol@sggw.edu.pl (M.S.); iga_piasecka@sggw.edu.pl (I.P.); diana_manko-jurkowska@sggw.edu.pl (D.M.-J.); agata_gorska@sggw.edu.pl (A.G.)

**Keywords:** pomegranate seed oil, ultrasound-assisted extraction, antioxidant activity, antioxidant stability, oxidation kinetics parameters, fatty acid profile

## Abstract

Pomegranate seed oil (PSO), a by-product of juice production, is rich in bioactive compounds, especially punicic acid, and has significant potential for health and industrial applications. The present study aimed to optimize an ultrasound-assisted extraction (UAE) of PSO and compare its effectiveness with conventional methods such as cold pressing and Soxhlet extraction. A Box–Behnken design was used to determine the optimal UAE parameters (amplitude 46%, 12 min, L/S ratio 19 mL/g), yielding 12.67% oil with the highest oxidative stability (τ_max_ = 5.44 min). Compared to Soxhlet and cold-pressed methods, UAE gave the highest yield, but slightly lower levels of total polyphenols and antioxidant activity. Cold-pressed oil retained the most bioactive compounds, but showed reduced oxidative stability and higher susceptibility to degradation. Soxhlet extraction provided moderate antioxidant capacity and the highest punicic acid content, but exceeded the recommended limits for acid value. Overall, the UAE offers an effective balance between yield, quality, and sustainability, with minimal thermal degradation and reduced solvent consumption. The results confirm that UAE is a promising alternative for high-quality PSO extraction, although cold pressing remains superior in preserving sensitive bioactive components. Ultimately, this study underscores that the extraction method plays a decisive role in determining the functional quality and oxidative stability of PSO, with UAE standing out as the most efficient and environmentally favorable approach.

## 1. Introduction

Fruit seeds are a significant by-product generated in large quantities during fruit processing. Accounting for approximately 20% of the fruit’s total weight, pomegranate seeds are a significant by-product of juice production. However, due to limited research and valorization, they contribute to an estimated annual waste of 70,000–100,000 tons [1,2].

Despite being a rich source of bioactive compounds, fruit seeds are often discarded as waste, leading to both the loss of valuable raw materials and an increased environmental burden [3,4,5]. However, scientific studies indicate that seeds, including those of pomegranates, contain essential nutrients and bioactive components that, when appropriately processed, can have diverse applications [6,7]. Among the most valuable products derived from pomegranate seeds is pomegranate seed oil (PSO). The extraction of PSO not only facilitates the utilization of this valuable resource but also aligns with the principles of a circular economy and the sustainable development strategy advocated by the European Union policy.

The oil present in pomegranate seeds is gaining increasing interest due to its unique fatty acid (FA) composition. PSO is distinguished by a high content of unsaturated fatty acids and conjugated fatty acids, among which the most significant is the isomer of α-linolenic acid—punicic acid (9-*cis*, 11-*trans*, 13-*cis* octadecatrienoic acid) [8,9]. Punicic acid (PA) is known for its diverse health benefits, including anticancer and anti-inflammatory properties. Studies indicate that PA exhibits selective cytotoxicity against cancer cells while sparing normal cells, highlighting its potential as a therapeutic agent [10]. In addition, PA has been shown to improve metabolic disorders by regulating the gut microbiota and reducing lipid accumulation in the liver [11]. Moreover, it has shown antiatherosclerotic and anti-diabetic effects and may be useful in combating insulin resistance and even in treating type 2 diabetes [12,13,14]. Furthermore, PSO is rich in bioactive compounds, mainly polyphenols and tocopherols, which exhibit antioxidant, anti-inflammatory, and antimicrobial properties. Polyphenols, mainly ellagic acid and anthocyanins, are antioxidants that help neutralize free radicals, reducing oxidative stress and inflammation [15]. The high levels of polyphenols and tocopherols in PSO provide potent antioxidant activity, crucial for preventing chronic diseases [15]. Additionally, the bioactive compounds in PSO have been shown to reduce inflammation and exhibit antimicrobial properties. Notably, PSO has effectively decreased mycotoxin secretion, highlighting its potential as a natural preservative in food systems [16]. The choice of oil extraction methods is critical to optimizing yield and preserving health-promoting components. Efficiently extracting PSO while preserving its bioactive components presents several challenges, as traditional methods such as solvent extraction and hot pressing can weaken the bioactive components of PSO due to thermal degradation [6].

Cold pressing is a method widely used in industry because of its ability to preserve bioactive compounds. However, compared to extraction with solvents, this method is much less efficient and more time consuming [17]. In addition, cold-pressed oils have a shorter shelf life. This is because they retain more natural compounds and do not contain chemical preservatives, and are more susceptible to oxidation and rancidity, requiring careful storage conditions [18]. Despite these limitations, cold pressing remains popular because of its environmental friendliness and the high quality of the oil that is produced, free of chemical residues. Soxhlet extraction is highly effective for extracting bioactive compounds from solid materials. Compared to cold pressing, in the Soxhlet method, more oil can be extracted due to the repeated recycling of solvent. However, prolonged exposure to heat can degrade thermolabile (heat-sensitive) compounds, affecting the quality of the extract [19]. Soxhlet extraction is identified as optimal for laboratory conditions, yielding high-quality oil, but is not widely used in industry, mainly due to oil contamination by residual solvent used in extraction [20].

Ultrasound-assisted extraction (UAE) is a technique that enhances the extraction of bioactive compounds from plant materials by utilizing ultrasonic waves. This method is known for its efficiency in breaking down cell walls and improving mass transfer, leading to higher yields of extracted substances. UAE works through the phenomenon of cavitation, in which ultrasonic waves create microbubbles in the solvent. These bubbles grow and implode, generating intense local pressure and temperature changes that break down cell walls, facilitating the release of intracellular compounds [21]. Implosion of cavitation bubbles increases solvent penetration into plant tissues, further aiding the extraction process [21]. Ultrasonic waves induce micro-acoustic fluxes and pressure differences around the particles, significantly increasing the mass transfer rate. UAE is distinguished by reduced energy consumption and minimal solvent consumption compared to conventional extraction methods. This makes it a more sustainable option for extracting bioactive compounds [22]. To maximize efficiency and yield, the process can be optimized by adjusting parameters such as ultrasound power, temperature, and solvent type [23].

The purpose of this study is to optimize the extraction process of PSO using UAE and to evaluate the effect of this method on FA composition, oxidative stability, quality parameters, and bioactive compound content compared to conventional oil obtaining methods such as cold pressing and Soxhlet extraction. The study aims to determine whether UAE is an effective alternative to conventional techniques in terms of the quality and health-promoting properties of the obtained oil.

## 2. Results and Discussion

### 2.1. Experimental Design—Ultrasound-Assisted Extraction

**Figure 1:**
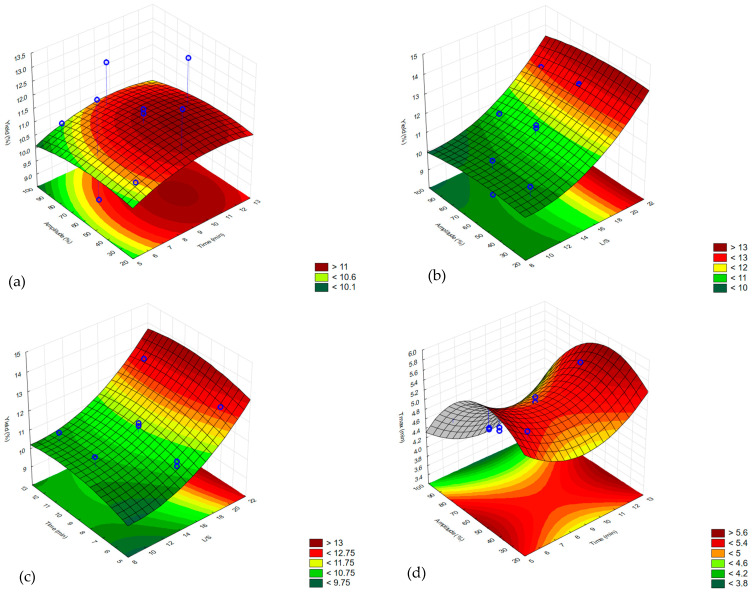

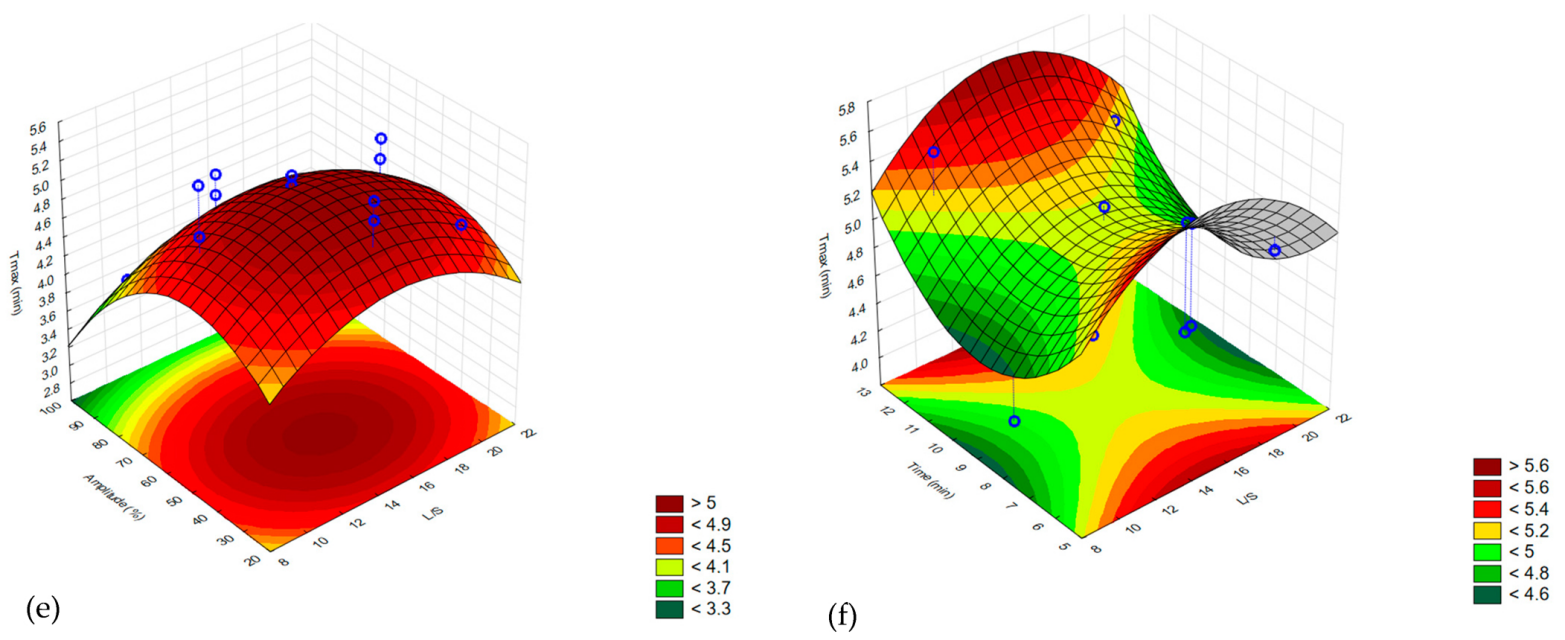
Effect of (**a**) extraction time and ultrasonic amplitude on oil yield at liquid to solid (L/S) ratio of 15; (**b**) ultrasonic amplitude and L/S ratio on oil yield at 9 min; (**c**) time and L/S ratio on oil yield at 60% ultrasonic amplitude; (**d**) extraction time and ultrasonic amplitude on maximum oxidation time (τ_max_) at L/S ratio of 15; (**e**) ultrasonic amplitude and L/S ratio on τ_max_ at 9 min; (**f**) time and L/S ratio on τ_max_ at 60% ultrasonic amplitude.

The UAE variables’ influence on yield and τ_max_ responses was analyzed. The suitable models and equations were fitted based on ANOVA results (Table 1). For the yield, a linear model was chosen as significant (*p* ≤ 0.05), and for τ_max_, a quadratic model (*p* ≤ 0.05) was preferred. Graphical expressions of the surface response are shown in Figure 1.

Generally, for the extraction yield, the only significant (*p* ≤ 0.05) variable was the L/S ratio. The observed relationship indicated that an increase in the L/S ratio corresponded to an enhancement in extraction yield, reaching the highest value of 12.85% in run number 8 (See section: Materials and Methods). The exact dependence is explained in Equation (1). The results obtained in previous findings are inconsistent in terms of factors influencing extraction yield. Tian et al. [24] also reported that the higher the L/S ratio, the higher the PSO yield could be obtained in the UAE process. According to the results obtained by Barizão et al. [25] in the UAE, L/S ratio, time, and temperature had a significant effect on PSO yield, however, the dependency of yield increase with increasing L/S ratio was confirmed only to some extent. When the L/S ratio reached a maximum of 28 mL/g, the yield was decreased compared to the results obtained for a 24 mL/g L/S ratio.

(1) Yield=35.77+0.33·Time−0.02·Amplitude+1.24·L/S 

In terms of maximum oxidation time (τ_max_), which represents resistance to oxidation of the oil sample, the square of time and amplitude, and also amplitude itself were identified as significant (*p* ≤ 0.05) variables. The results of τ_max_ were in a range of 3.95–5.47 min. Rather longer sonication (12 min) accompanied by mild amplitude values (60%) resulted in longer τ_max_. Based on the results, the adequate model described by Equation (2) was fitted. Maximum oxidation time has not been assessed in the optimization studies for UAE of pomegranate seed oil yet. However, PSO is recognized as prone to oxidation reactions due to a high abundance of unsaturated fatty acids [26], and any improvement noted with UAE application is remarkable. As the UAE enables obtaining the products in a short treatment time, its application may prevent oil degradation when compared to traditional solid–liquid extraction. It is highly important, especially regarding oil with a significant amount of polyunsaturated fatty acids (PUFAs). Also, the lower temperature of the UAE prevents bioactive compounds from deteriorating. However, if the sonication is too intense or too long, it may result in peroxide formation and promote the oxidation of oils. That is why there is a need to optimize the UAE procedure to find the most effective extraction conditions that do not cause extensive peroxide formation [27].

(2) $$\tau_{max}=3.39-0.59\cdot Time-0.06\cdot Amplitude+0.40\cdot L/S-0.00008\cdot Time\;\;\cdot Amplitude-0.005\cdot Time\cdot L/S-0.003\cdot Amplitude\cdot L/S\;\;+0.04\cdot {Time}^{2}-0.0005\cdot {Amplitude}^{2}+0.01\cdot {L/S}^{2}$$

The results were analyzed in ANOVA to obtain the most effective conditions. The most optimal process parameters were selected to maximize both extraction yield and maximum oxidation time (τ_max_) to the greatest extent possible. The extraction conditions were set within the range proposed in the experiment, specifically an amplitude of 30–90%, 6–12 min durations, and an L/S ratio of 10–20. The software proposed 38 solutions, and one with the highest desirability was chosen. The exact values were 45.82% amplitude, 12 min time, and 18.88 L/S ratio. To adjust the proposed variables, the real applied parameters were 46% amplitude, 12 min time, and 19 L/S ratio. The predicted responses of yield and τ_max_ were 12.43% and 5.30, respectively. The mean yield result was 12.67 ± 0.48%, which differs from the predicted value by only 1.9%. For the τ_max,_ the actual mean value of 5.44 ± 0.02 min was recorded, which deviates from the predicted one by 2.6%. The results indicate that the model and equation predicted the results well. 

### 2.2. Oil Yield

Each obtaining method has unique advantages and efficiencies, affecting the quality and composition of the final oil. The yield of the process of obtaining PSO varied significantly depending on the applied extraction method (Table 2).

The lowest yield was observed for cold-pressed oil (CP_PSO), with a recovery of 9.61 ± 0.22%. The Soxhlet extraction method (SE_PSO) exhibited a higher yield, reaching 11.49 ± 0.19%, indicating its greater efficiency compared to cold pressing. The highest yield was obtained using ultrasound-assisted extraction, with a value of 12.67 ± 0.48%. The superior efficiency of the Soxhlet and UAE methods may be attributed to the enhanced lipid extraction in the presence of solvents and/or the application of more intensive process conditions, such as elevated temperature and ultrasound treatment. In contrast, the lower yield of cold pressing suggests that although this method is more beneficial for preserving bioactive compounds, it is less efficient in terms of oil recovery.

The obtained extraction yields are consistent with previous literature reports, which highlight the significant impact of extraction parameters on the amount of oil recovered from pomegranate seeds. In the study by Dib et al. [28], the Soxhlet extraction yield of PSO using *n*-hexane as a solvent ranged from 15.62% to 16.25%, which is slightly higher than the yield obtained in this study. Similarly, in the study by Tian et al. [24], the Soxhlet extraction yield was reported as 20.50%. Regarding cold pressing, the research by Kaseke et al. [29] reported a yield of 3.20%, which is approximately three times lower than the yield observed in this study. Furthermore, previous studies on the optimization of pomegranate seed oil extraction using UAE reported higher yields than those obtained in the present study, reaching 25.11% [24] or 27.99% [25].

However, the content of oil in the pomegranate seeds may be influenced by the origin of the seeds, the cultivar, and moisture content. In the case of cold pressing, the efficiency of oil extraction may also depend on the oil press equipment used in the process [29].

### 2.3. Fatty Acid Profile of Oils

In vegetable oils, the predominant group of lipids are triacylglycerols (TAGs). Both the composition and distribution of FA in TAG affect the nutritional properties and stability of the oil. Table 3 shows the FA profile of PSO obtained by different methods. Regardless of the method of oil extraction, the same FAs in similar contents were identified. In PSO, saturated fatty acids (SFAs) accounted for approximately 6%, while monounsaturated fatty acids (MUFAs) contributed around 5–6%. The FA composition of PSO was dominated by PUFAs, especially punicic acid (C18:3 n-5), and its isomers, which together accounted for approx. 85%. The slightly lower content of PA in the UAE-treated samples (78.68%) compared to the Soxhlet (79.15%) ones and significantly lower than in cold-pressed (80.80%) samples, despite a higher extraction yield, may suggest the occurrence of subtle structural modifications, such as partial degradation or isomerization, as a result of ultrasonic treatment. Although the observed difference is relatively minor and may fall within the range of analytical or biological variability, it is consistent with previous studies reporting that high-energy ultrasound can affect the integrity of conjugated systems under certain conditions. In particular, thermolabile FAs like PA may be susceptible to changes induced by localized hotspots, shear forces, and the formation of free radicals during sonication, potentially leading to the generation of positional or geometrical isomers, or even oxidative degradation products [30,31].

The highest percentage of PUFAs was obtained for oil extracted using the Soxhlet method, and the lowest for oil obtained using ultrasound treatment.

The literature data indicate that PA is the characteristic FA of PSO, accounting for 55 to 81% of all FAs. The composition of FAs (including the content of PA) can vary depending on the pomegranate variety, the place it was grown, the time it was harvested, or the method the oil was obtained [32]. Rowayshed et al. [33] studied PSO extracted using petroleum ether in a Soxhlet apparatus, which included only 59.40% PA. Habibnia et al. [34] showed that the oil, extracted from five different Iranian pomegranate seed varieties using *n*-hexane as the solvent at low temperatures, contained between 78.25 and 82.40% PA. Their data were similar to those obtained for PSO in the present study. Dadashi et al. [35] also extracted oil using *n*-hexane at room temperature, and they determined lower PA contents (from 72.07% to 73.31%) in the PSO tested than Habibnia et al. [34]. Juhaimi et al. [36] extracted oil from six fruit varieties of pomegranate picked from the Mediterranean region in Turkey, including the Hicaz one, as in the present study. A unique PA was found in pomegranate seed as the dominant FA in the range from 71.17 to 77.62%, while the highest value was noted for the Hicaz variety. Eikani et al. [37] compared superheated hexane extraction (SHHE), Soxhlet extraction, and the cold-pressing method to extract PSO. The FA profile for SHHE was more similar to that obtained by the cold-pressing method. Moreover, the percentage of PA was significantly lower after SHHE or cold pressing (70.73 and 69.79%, respectively) than after Soxhlet extraction (81.69%). Such significant differences in the percentage content of PA, depending on the method of oil obtaining, were not noted in the present study.

### 2.4. Quality Parameters of Oils

The acid value (AV) determines the amount of free fatty acids in fat and is expressed in mg KOH per 1 g of fat. According to the standards established by the Codex Alimentarius [38], AV should not exceed 4 mg KOH/g of fat in cold-pressed oils. The peroxide value (PV) is a measure of the peroxide content, which is the primary product of lipid oxidation, and indicates the degree of oxidation or rancidity of fat. It is expressed in milliequivalents of active oxygen per 1 kg of fat. The PV for cold-pressed oils is specified in the EN ISO 3960:2017-03 standard [39], which states that it should not exceed 10 meq O_2_/kg, while the Codex Alimentarius [38] sets the limit at 15 meq O_2_/kg. Table 4 shows the quality parameters of the extracted PSO. 

The results indicate that cold-pressed PSO exhibited the lowest AV among the tested samples (2.23 mg KOH/g). The highest AV was observed for PSO obtained by the Soxhlet method (4.75 mg KOH/g), which exceeded the recommended standard. Contrarily, the lowest PV was recorded for oil extracted using the Soxhlet method (3.20 meq O_2_/kg), while all tested oils complied with the 10 meq O_2_/kg fat limit established by EN ISO 3960:2017-03 [39]. The PV of cold-pressed PSO (4.68 meq O_2_/kg) was the highest among the tested oils.

The literature data show a wide range of AV and PV obtained for PSO, as they depend on many factors, including the origin and quality of the raw material, its cultivation conditions, methods, and parameters of oil production or storage conditions. Khoddami and Roberts [40] reported that the PV of cold-pressed PSO ranged from 4.67 to 5.96 meq O_2_/kg fat, and the AV value of CP_PSO (4.68 meq O_2_/kg fat) falls within this range. Meanwhile, Juhaimi et al. [36] obtained higher PV values (5.44 to 8.47 meq O_2_/kg fat) for PSO and Costa et al. [41]—lower ones (0.91–2.69 meq O_2_/kg fat). For the commercial PSO studied by Melo et al. [8], the AV (0.63 mg KOH/g fat) was significantly lower than for PSO presented in this study, but the PV (3.34 meq O_2_/kg) was close to the PV of SE_PSO (3.20 meq O_2_/kg). In turn, Dadashi et al. [35] determined AV values ranging from 3.78 to 8.36 mg KOH/g fat for Soxhlet-extracted PSO, similar to the AV of SE_PSO (4.75 mg KOH/g fat) in the present study but at the same time—much lower PV (0.39–0.50 meq O_2_/kg fat).

During storage, peroxides can undergo further transformations, leading to the formation of secondary oxidation products such as aldehydes or ketones, which affect undesirable changes in the taste, odor, and nutritional value of the oil. The anisidine value (p-AnV) determines the content of secondary oxidation products, primarily aldehydes. It is considered a more sensitive indicator of advanced fat oxidation than PV. Considering the level of secondary oxidation products, comparable results were obtained for extracted oils and higher for cold-pressed PSO. Also, the TOTOX index, which provides information on both early and advanced stages of fat oxidation, was the highest for CP_PSO (25.14). These findings suggest that cold-pressed oil may be more prone to oxidation, likely due to enhanced exposure to oxygen during processing.

In the study by Ghorbanzadeh and Rezaei [42], cold-pressed PSO exhibited a significantly lower p-AnV (5.11) and TOTOX index (11.61) compared to PSO obtained by the same method in the present study. Conversely, the p-AnV (17.17) and TOTOX (26.7) values for oil extracted using the Soxhlet method were slightly higher than those obtained in this study [42]. The results obtained for PSO extracted by the Soxhlet method in the present study demonstrated higher p-AnV and TOTOX compared to the findings reported by Alaşalvar et al. [43], in which the p-AnV was 8.54, and the TOTOX index reached the value of 17.52. Although the UAE is increasingly employed for the extraction of plant oils due to its high efficiency, shortened processing time, and environmental friendliness, the scientific literature still lacks comprehensive data concerning such quality indicators as p-AnV and TOTOX index for PSO extracted using this method.

Higher values of PV, p-AnV, and TOTOX in cold-pressed PSO compared to those obtained by Soxhlet extraction or UAE can be due to several factors: (1) cold-pressed oil is obtained through mechanical pressing at low temperature without any refining step, therefore, it can contain not only naturally occurring antioxidants but also more oxidation-prone compounds, which can contribute to higher PV and p-AnV; (2) cold pressing involves prolonged contact with air, leading to oxidation of PUFA; (3) Soxhlet extraction and UAE use organic solvents or cavitation effects, respectively; therefore, they could provide better lipid solubilization and reducing oxidation by limiting contact with air during the process; (4) UAE operates under controlled conditions that minimizes oxidation. The observed differences in quality parameters suggest that the method of fat obtaining may influence the oxidative stability of PSO.

### 2.5. Oxidation of Oils

#### 2.5.1. Oxidative Stability

Oxidative stability, or oil’s resistance to oxidation processes, is a key parameter of edible oils that determines their quality, shelf life, nutritional value, and suitability for consumption and processing [44]. The pressure differential scanning calorimetry (PDSC) method provides a rapid and reproducible assessment of lipid oxidation in the selected oil under accelerated conditions, making it a highly effective tool for oxidative stability analysis. This technique enables the real-time recording of oxidation changes in a PDSC oxidation curve (heat flow changes as a function of time), allowing for precise characterization of the oil’s oxidative behavior, e.g., for determining oxidation-related parameters like oxidation induction time (OIT). OIT is the time from the initial exposure to oxygen to the onset of exothermic decomposition at the isothermal conditions of the test. It is known that the longer the OIT is, the more stable the oil is [45].

Based on the obtained PDSC oxidation curves, both OIT and the oxidation time corresponding to the maximum heat flow values (to the maximum intensity of oxidative changes occurring in the sample; τ_max_) were determined and shown in Table 5. OIT and τ_max_ decreased with increasing oxidation temperature, regardless of the method in which the oil was obtained. It is a well-known tendency, as, according to van ’t Hoff’s empirical rule, when the temperature increases by approximately 10 K, the reaction rate increases approx. 2–4 times [46]. The present study indicated that increasing the temperature by 10 °C between 90 and 120 °C reduced OIT by about 2.6 to 3 times. The temperature coefficient, which determines how many times the reaction rate increases when the temperature is raised, was the highest for cold-pressed PSO. Oxidative changes also began much faster in cold-pressed PSO (e.g., ~60 min. at 90 °C) than in oil extracted by the Soxhlet method or by ultrasound (~105–107 min. at 90 °C). As was mentioned earlier, cold pressing is a mechanical process that does not involve using solvents or high temperatures; therefore, the obtained oil can contain more impurities, including enzymes, trace metals, phospholipids, free fatty acids, and moisture, than oil after Soxhlet extraction. Such impurities can act as oxidation catalysts or pro-oxidants and accelerate oxidation. However, in the present study, it was shown that the AV value of CP_PSO was lower than that of SE_PSO; therefore, the amount of free fatty acids was lower in cold-pressed PSO. In turn, SE_PSO was characterized by lower PV, p-AnV, and TOTOX than other tested oils. It can be stated that the oil has undergone hydrolytic breakdown rather than oxidative damage. Cold-pressed oil preserves natural enzymes from the seeds, such as lipoxygenase and peroxidase, which can accelerate lipid oxidation; meanwhile, the Soxhlet method may inactivate these enzymes, leading to slower oxidation rates. Moreover, cold pressing preserves all naturally occurring compounds, but some of them may contribute to pro-oxidation reactions when exposed to oxygen, while Soxhlet extraction can selectively remove some oxidation-prone minor compounds, increasing overall stability. It is worth mentioning that cold-pressing exposes the oil to oxygen and friction-generated heat, which initiates oxidation at an early stage, but Soxhlet extraction occurs in a closed system with a solvent, minimizing direct exposure to oxygen and delaying the onset of oxidation.

A study by Yoshime et al. [47] showed that PSO was characterized by a shorter OIT than other edible oils. According to the authors, it was related to the high concentration of PA, which dominated the composition of FA. It is known that a significant number of multiple bonds increases the rate of oil oxidation; for example, linoleic acid oxidizes from 10 to 40 times faster than oleic acid, whereas α-linolenic acid oxidizes 2–4 times faster than linoleic acid [48]. PA with three double bonds is an isomer of α-linolenic acid; however, in PA, these double bonds are conjugated rather than methylene interrupted, which alters its reactivity. Ratusz et al. [49] analyzed the autooxidation process of model samples of refined vegetable oils, such as rapeseed and sunflower, using PDSC. The oxidative stability was determined at 120 °C according to the methodology used in the present paper. Their results showed that OIT for rapeseed oil was 56.73 min, while for sunflower oil, it reached 24.55 min. Since PSO belongs to the group of specialty oils, the stability of PSO was also compared with data available in the literature for such kind of oil, e.g., watermelon seed oil. The lowest τ_max_ value for unrefined cold-pressed watermelon seed oil (23.88 min) determined at 120 °C by Siol et al. [50] was more than 7× higher than for CP_PSO (3.37 min). In contrast, the authors obtained the highest τ_max_ value for watermelon seed oil extracted with hexane at room temperature (76.55 min). The difference between τ_max_ values for cold-pressed and extracted watermelon seed oil was twice as much as for PSO determined in this study at the same temperature. Most likely, depending on whether it is the FA composition or the antioxidant content that determines to a greater extent the oxidative stability of a given oil, its method of extraction may have a greater or lesser effect on the observed differences in oxidative stability.

#### 2.5.2. Oxidation Kinetics

The oxidation process in accelerated methods takes place at high temperatures and has a different course than at ambient temperature. However, determining the stability of oils at different temperatures allows for determining the parameters of the oxidation kinetics, which helps estimate the oil’s durability [46]. The scientific literature still lacks comprehensive data concerning the oxidation kinetics of PSO.

The oxidation of oils is a multi-step reaction that typically proceeds through a free radical mechanism. According to Kowalski et al. (2004), the oxidation of seed oils in excess of oxygen follows a first-order reaction mechanism [51]. However, it should be noted that oxidation of oils is theoretically not a first-order reaction, but such a model can be used as an approximation in kinetic analysis. Kaseke et al. (2021) fitted different kinetic models to evaluate lipid oxidation kinetics, and they selected the first-order one as the best fitting reaction order [52].

Determination of the kinetics of vegetable oil oxidation was primarily related to the analysis of the activation energy (*E*_a_) and the reaction rate constant (*k*). The results of the calculations of kinetic parameters are listed in Table 6. The τ**_max_** measured under isothermal conditions (90–120 °C) using the PDSC method allowed for the preparation of a graphical relationship between the τ**_max_** logarithm and the reciprocal of temperature. R^2^ coefficients above 0.99 meant that the data describing linear correlations could be used for further calculations. The *E*_a_ of the oxidation reaction for oil samples extracted from pomegranate seeds ranged from 106.15 to 111.99 kJ/mol. Kaseke et al. (2021) determined the E_a_ for the lipid oxidation reaction in PSO extracted from unpretreated, blanched, and microwaved seeds as equal to 6.29, 8.98, and 8.59 kJ/mol, respectively. However, the authors used a different method to study oxidation kinetics using peroxide values to assess the oxidative stability of PSO samples stored at 50 °C [52]. The E_a_ values depend on the measurement method used as well as the experimental conditions. In the PDSC method, pure oxygen under pressure is applied, which may lead to higher energy activation values.

Similar to the results in the present paper, the studies by Wirkowska et al. [53] showed a strong inverse relationship between temperature and τ_max_, which enabled the determination of kinetic parameters due to the high agreement of the linear model (R^2^ > 0.99). Amaranth and quinoa oils were the subject of the scientists’ research. It was found that the *E*_a_ of oxidation of amaranth and quinoa oils was lower than in the case of linseed, camelina, walnut, olive, and rapeseed oils, which may be related to the higher content of unsaturated FA in these oils.

The *E*_a_ of the oxidation reaction for linseed oils studied by Symoniuk et al. [54] was lower than for the samples analyzed in this study and ranged from 93.14 to 94.53 kJ/mol, while for rapeseed oil, according to Symoniuk et al. [55], *E*_a_ ranged from 86.71 to 90.54 kJ/mol. The *E*_a_ values obtained in the present study were also higher than those determined by Ciemniewska-Żytkiewicz et al. [56] for hazelnut oil (89.06 kJ/mol), rapeseed oil (92.68 kJ/mol), and olive oil (92.81 kJ/mol), and by Ratusz et al. [44], for camelina oil (87.63–93.61 kJ/mol). In turn, Farhoosh et al. [57] reported activation energy values for corn oil—88.14 kJ/mol, sunflower oil—90.24 kJ/mol, and soybean oil—92.42 kJ/mol, which were also lower than those obtained in this study.

Taking into account the literature data, *E*_a_ values exceeding 100 kJ/mol can be considered high. Despite the significant content of unsaturated FA in PSO, which promotes oxidation processes, it was observed that more energy was required to initiate the reaction than in many other oils. PSO, although it may exhibit high *E*_a_ under controlled conditions, is characterized by low oxidative stability due to the presence of highly reactive PA. Once oxidation is initiated, the process proceeds rapidly, making the oil very sensitive to oxygen, light, and heat.

The oxidative stability of vegetable oils depends not only on their FA composition, but also on the presence of natural antioxidant compounds, which can significantly slow down degradation processes. In the study conducted by Song et al. [58], the kinetic parameters of oxidation of different oils at 180 °C were compared using the Ozawa–Flynn–Wall methods and the Arrhenius equation. It was shown that the presence of natural or synthetic antioxidants increased the *E*_a_ and decreased the value of *k*, which resulted in a slower oxidation process. In this study, comparing different extraction methods, it was found that the oil obtained by sonication was characterized by the highest *E*_a_ value, which may indicate its greater oxidation resistance. Similar results were obtained in the study by Piasecka et al. [59], which aimed to determine the optimal conditions for the extraction of cranberry seed oil using UAE and to assess the effect of this method on the properties of the final product. Based on PDSC measurements carried out at five temperatures, the kinetic parameters of the oxidation reaction were calculated, and, similarly to this study, a higher *E*_a_ value was found for the oil extracted by the method assisted by ultrasounds than for oil obtained by the conventional solid–liquid extraction with *n*-hexane. It is worth noting that the results of Piasecka et al. [59] partially contrast with the reports of Pérez-Sauced et al. [60], who showed that the use of ultrasound for the extraction of avocado oil resulted in a lower *E*_a_ compared to cold extraction by centrifugation. These differences confirm that the extraction method and its conditions have a significant impact on the oxidative stability of the obtained oils, and the effect of ultrasound may depend on the type of raw material, process parameters, and chemical composition of the oil.

Considering the reaction rate coefficients obtained in this paper, it can be stated that they increased with increasing temperature. For example, at 90 °C, the rate coefficient value was from 0.0009 to 0.009 min^−1^, while at 120 °C, it was from 0.0145 to 0.0247 min^−1^. In the study by Symoniuk et al. [54] it was shown that *k* for linseed oil, calculated based on the results obtained by the PDSC method at 100 °C, were higher than the values presented by Ciemniewska-Żytkiewicz et al. [56] for hazelnut oil (0.0071 min^−1^), olive oil (0.0066 min^−1^), and rapeseed oil (0.0108 min^−1^), and ranged from 0.0636 to 0.0890 min^−1^. These results confirm the greater susceptibility of linseed oil to oxidation. For comparison, *k* for the tested PSO samples at the same temperature (100 °C) ranged from 0.0024 to 0.0043 min^−1^, which indicates their moderate susceptibility to oxidation.

### 2.6. Bioactive Properties

PSO is a valuable source of numerous bioactive compounds that confer significant health-promoting properties. Particular attention is given to its high content of conjugated FAs, primarily PA, which constitutes the dominant lipid fraction in the oil and belongs to the group of conjugated linolenic acids (CLnA). This compound has well-documented anticancer and anti-inflammatory activities [12,61,62]. PSO also contains a variety of phenolic compounds, which are key contributors to its free radical scavenging capacity. These phenolics support cellular defense mechanisms against oxidative stress, thereby delaying cellular aging and degeneration processes [36]. Other important bioactive constituents include tocopherols (mainly γ- and δ-tocopherol), which act as natural lipid-soluble antioxidants; phytosterols (such as β-sitosterol and campesterol), known for their hypolipidemic and anti-inflammatory effects [47]; and flavonoids, which further enhance antioxidant activity through synergistic interactions with other bioactive components present in PSO [41].

The study demonstrated that the extraction method influenced the content of bioactive compounds in PSO (Table 7). Oil extracted using the Soxhlet method contained fewer polyphenols compared to other tested oils due to the high temperature and prolonged extraction times involved in the process. Some polyphenols are heat sensitive, and they tend to degrade or remain in the seeds during solvent extraction. Additionally, the use of organic solvents may not be as selective for polyphenols compared to other, gentler extraction methods like cold pressing or UAE. Cold-pressed oil exhibited a significantly higher polyphenol content than oil extracted using the Soxhlet method and oil obtained through UAE. The polyphenol content in CP_PSO was approximately 1.7 times higher than in SE_PSO and 1.2 times higher than in UAE_PSO. These findings are consistent with previous research, which indicates that non-thermal extraction methods, such as cold pressing, more effectively preserve thermolabile polyphenolic compounds than solvent-based methods [29]. However, it should be noted that cold-pressed oil, although it had the highest polyphenol content, also had the lowest oxidative stability (Table 5 and Table 7). The cold-pressing process did not include refining steps that could remove pro-oxidative compounds. These steps help eliminate free fatty acids, phospholipids, pigments, minerals, metals, and other pro-oxidative substances that can accelerate oxidation. Although refining also reduces the content of natural antioxidants, the removal of oxidation-promoting compounds often results in a more oxidatively stable oil compared to cold-pressed oil. Unrefined cold-pressed oils are more vulnerable to degradation when exposed to air, light, or heat—even though they contain a high level of antioxidants.

In the study by Khoddami et al. [63], oil from three different pomegranate seed varieties was cold-pressed, and the reported total polyphenol content (TPC) ranged from 8.52 to 10.44 mg GAE/g PSO, which is approximately four times higher than the values obtained in the present study. In contrast, in the study by Abbasi et al. [64], PSO extracted using the Soxhlet method was characterized by TPC of 9 mg GAE/g oil. The study by Juhaimi et al. [36] demonstrated that Soxhlet extraction yielded oil with TPC ranging from 7.8 to 19.2 mg GAE/g. TPC of pomegranate seed oil from the Hicaz variety was found to be 8.71 mg GAE/g [36], which is significantly higher than the value obtained in this study. On the other hand, the TPC values observed in this study are significantly higher than those reported for PSO from the Tounsi variety (93.42 mg GAE/kg) [65].

Antioxidant activity determined using the ABTS method indicates that cold-pressed oil exhibits the highest capacity for neutralizing free radicals. A similar trend was observed in antioxidant activity measured using the DPPH method.

Cold-pressed pomegranate seed oil [29] showed markedly higher ABTS and DPPH radical scavenging activity compared to the oil examined in the present study, indicating substantial differences in their antioxidant potential. In the study by Rojo-Gutiérrez et al. [66], the ABTS and DPPH radical scavenging activity of PSO obtained using UAE was significantly higher than the values obtained in this work. Few available scientific publications focus specifically on the determination of the antioxidant activity of PSO. In the majority of studies concerning pomegranate seeds, antioxidant activity values measured by ABTS and DPPH assays are reported in relation to ethanolic, acetonic, or aqueous extracts, or expressed per unit of dry seed mass [67,68,69]. Consequently, there is a need for further research aimed at standardizing analytical methodologies specific to PSO, to enable reliable comparisons across different sample types and extraction methods.

Differences in TPC and antioxidant activity of PSO between various extraction methods can be attributed to variations in solvent selectivity, as well as the applied temperature and extraction duration. The higher TPC and antioxidant potential of cold-pressed oil may be due to the absence of heat exposure, which protects phenolic compounds from degradation. Conversely, the lower efficiency of Soxhlet and UAE extraction is likely a result of thermal and solvent-induced degradation of bioactive compounds [70,71]. Additionally, the observed differences may arise from the varying ability of each method to extract hydrophilic and lipophilic bioactive substances—while the ABTS method measures both hydrophilic and lipophilic antioxidants, the DPPH method primarily detects lipophilic compounds [72,73]. Furthermore, the content of bioactive compounds and antioxidant activity in fruit seed oils is influenced by factors such as origin, variety, ripening stage, and seed quality [74]. Storage and transportation conditions of the seeds may also play a crucial role. It is important to note that the analyzed oils were extracted from seeds that were a by-product of juice pressing rather than directly from fresh fruit seeds.

## 3. Materials and Methods

### 3.1. Materials

The pomegranate seed oils were obtained from raw materials (*Punica granatum* L. var. Hicaz) grown in Croatia. After juice production, the pomegranate seeds were dried in the sun and transported to Poland in airtight, sterile thermal containers to protect them from moisture and light. The Folin–Ciocalteu reagent, DPPH reagent, ABTS reagent, and *p*-anisidine were purchased from Sigma-Aldrich (USA), and other chemicals were purchased from Chempur (Poland).

### 3.2. Methods

#### 3.2.1. Oil Obtaining Methods

##### Cold Pressing

Oil was collected from 200 g of pomegranate seeds using an automated oil cold-pressing machine YODA YD-ZY-02A (Yoda, Warsaw, Poland). The temperature program was set in the range of 32–38 °C. The pressing time was around 8 min. The oil was carried down to a decantation vessel. Extraction was performed three times. The oil was collected, separated by centrifugation, and stored at 4 °C before testing.

##### Soxhlet Extraction

To obtain the oil by hexane extraction using a Soxhlet apparatus, 20 g (±0.001 g) of seeds was weighed and ground in an IKA Tube Mill (IKA Works GmbH & Co. KG, Staufen, Germany) at 25,000 rpm. The seeds crushed in this way were wrapped in filter paper, put into a thimble, and placed in a Soxhlet apparatus. Hexane (200 mL) was added to the round-bottom flask. The extraction was carried out for 6 h. The obtained extracts were dried with anhydrous magnesium sulfate, which was then filtered off. The solvent was distilled using a vacuum rotary evaporator Rotavapor^®^ R-300 (BUCHI, Uster, Switzerland). Residual *n*-hexane was removed from oil samples in nitrogen atmosphere.

##### Experimental Design—Ultrasound-Assisted Extraction

Based on previous PSO optimization studies [19,20,26], the variables were chosen for a three-factor, three-level Box–Behnken design of experiment: ultrasound amplitude in a range of 30–90%, sonication time in a range of 6–12 min, and L/S ratio in a range of 10–20. Detailed variables of all performed runs are shown in Table 8. The responses chosen for the experiment were oil yield (%) and maximum oxidation time (τ_max_). Then, suitable models were fit according to ANOVA results. Specific equations describing models were also determined (3- for the linear model and 4- for the quadratic model):

(3) $$Y=\beta_{0}+\beta_{1}X_{1}+\beta_{2}X_{2}+\beta_{3}X_{3}$$

(4) $$Y=\beta_{0}+\beta_{1}X_{1}+\beta_{2}X_{2}+\beta_{3}X_{3}+\beta_{12}X_{1}X_{2}+\beta_{13}X_{1}X_{3}+\beta_{23}X_{2}X_{3}+\beta_{11}{X_{1}}^{2}\;+\beta_{22}{X_{2}}^{2}+\beta_{33}{X_{3}}^{2}\;$$

where $\beta_{0}—$the constant coefficient; $\beta_{1}$, $\beta_{2}$, $\beta_{3\;}$—regression coefficients for the linear terms; $\beta_{11}$, $\beta_{22}$, $\beta_{33}$—regression coefficients for the quadratic terms; $\beta_{12}$, $\beta_{23}$, $\beta_{13}—$regression coefficients for the interaction terms; $X_{1}$, $X_{2}$, $X_{3}—$coded values of independent variables.

##### Ultrasound-Assisted Extraction

UAE of the oils was carried out in a UP200Ht ultrasonic processor supplied by Hielscher Ultrasonics GmbH (Tetlow, Germany) with an output power of 200 W, according to the method proposed by Piasecka et al. [75] with minor modifications. Seed samples (5 ± 0.001 g) were ground using an IKA tube mill (IKA-Werke, Staufen im Breisgau, Germany) at 20,000 rpm for 60 s, then placed in a Falcon tube and filled with *n*-hexane at a proper L/S ratio just before extraction. The resulting extracts were filtered, dried with anhydrous magnesium sulfate, and evaporated at 70 mbar. The remaining *n*-hexane was removed under a nitrogen atmosphere. The parameters chosen as optimal process conditions were 46% amplitude, 12 min, and 19 L/S ratio.

#### 3.2.2. Oil Yield Determination

The oil yield obtained from pomegranate seeds was measured using a gravimetric test, calculated by dividing the oil’s weight by the seeds’ initial weight and expressed as a percentage, according to Equation (5) [76].

(5) $$Yield\;[\%]=\frac{m_{o}}{m_{s}}\times 100$$

where m_o_—mass of oil; m_s_—mass of seeds.

#### 3.2.3. Determination of Fatty Acid Composition by Gas Chromatography

A gas chromatograph YL6100 GC (Young Lin Bldg., Anyang, Hogyedong, Republic of Korea) equipped with a BPX-70 capillary column (SGE Analytical Science, Milton Keynes, UK) with the following parameters: length: 60 m; film thickness: 0.25 μm; and inner diameter: 0.25 mm; and a flame ionization detector was applied to determine the composition of fatty acids present in the oils. The fatty acid methyl esters (FAME) were prepared according to PN-EN ISO 5509:2001 [77]. The gas that carries FAME inside the column was nitrogen. The injector and detector temperatures were set at 225 °C and 250 °C, respectively. The temperature program for the GC started at 70 °C, held for 0.5 min, then increased at (I) 15 °C/min to 160 °C; (II) 1.1 °C/min from 160 °C to 200 °C (held for 12 min) and (III) 30 °C/min from 200 °C to 225 °C.

The individual FAs were identified by comparing the retention times of FAME. The abundance percentage of each FA was also determined. The determinations were performed at least in duplicate.

#### 3.2.4. Determination of Acid, Peroxide, and p-Anisidine Values and TOTOX Index

The degree of hydrolysis of the tested oils was determined using AV according to the AOCS Cd 3d-63 [78]. The content of primary oxidation products of the oils was examined by PV according to the AOCS method (AOCS Cd 8–53) [79]. The degree of secondary oxidation of oils was determined by p-AnV according to the AOCS method (AOCS Cd 18–90) [80]. The TOTOX index, indicating the total oxidation value, was calculated according to the formula: TOTOX index = (2 × PV) + p-AnV.

#### 3.2.5. Determination of Oxidative Stability and the Oxidation Kinetics Using the PDSC Method

Pressure differential scanning calorimetry using DSC Q20 TA Instruments (Newcastle, WA, USA) was employed to determine the OIT and τ_max_ as well as kinetic parameters for the oxidation reaction of oils. The weight of the oils used in the tests ranged from 3 to 4 mg. Oil placed in an open aluminum pan was oxidized at a pressure of ~1400 kPa in a cell with an empty reference pan. The samples were tested under isothermal conditions. The accelerated oxidation of oil samples in a PDSC cell was set at temperatures of 90, 100, 110, and 120 °C.

The OIT was determined as the intersection of the extrapolated baseline and the tangent line (leading edge) of the exothermic curve and was measured in minutes, while τ_max_ was determined as the time corresponding to the maximum of the PDSC signal.

The isothermal PDSC variant was employed to assess oxidation kinetic parameters by using the following Equations (6)–(8) [59] and according to the Ozawa–Flynn–Wall methodology:

(6) $$log\tau_{max}=a\cdot \left(\frac{1}{T}\right)+b\;$$

where $\tau_{max}\;$is maximum oxidation time (min) and *T* is the temperature (*K*);

(7) $$E_{a}=2.19\cdot R\cdot a$$

where *E_a_*—activation energy, *R* (J·mol^−1^·K^−1^) is a gas constant, and *a* is a coefficient from Equation (6);

(8) $$k=Ze^{\frac{{-E}_{a}}{RT}}$$

where *k* is a reaction rate coefficient (min^−1^); Z is a pre-exponential factor.

#### 3.2.6. Preparation of Extracts for Analysis of Bioactive Properties of Oils

A total of 0.5 g of oil was mixed with 2.5 mL of hexane and 2.5 mL of methanol. The mixture was then shaken for 2 min, centrifuged (4000 rpm) for 10 min, and the bottom layer was collected in a new tube. The procedure was repeated 3 times.

#### 3.2.7. Determination of Total Polyphenol Content (TPC)

The method of determination proposed by Gao et al. [81] was used to determine the content of total polyphenols. In test tubes, 200 µL of sample, 0.3 mL of Folin–Ciocalteu reagent, as well as 4.9 mL of distilled water and 0.6 mL of 20% sodium carbonate solution were measured sequentially. The contents of the tubes were mixed using a Vortex centrifuge and left in a dark place for 60 min. After this time, the absorbance at λ = 765 nm was measured against the blank, which was methanol, using a Jenway 6305 UV–VIS spectrophotometer (Cole-Parmer, Vernon Hills, IL, USA). The calibration curve was prepared using working standard solutions of gallic acid at concentrations ranging from 100 mg to 1200 mg/L. TPC values were given in milligrams of gallic acid equivalent (GAE) per 1 g of oil.

#### 3.2.8. Determination of Antioxidant Activity Using ABTS Cation Radicals

To determine the antioxidant activity of the extracts obtained from the oils, the method described by Re et al. [82] with modifications was used. For this purpose, an ABTS (2,2′-azino-bis-(3-ethylbenzothiazoline-6-sulfonic acid) solution was prepared by mixing 14 mM ABTS solution with 4.9 mM of potassium persulfate solution in a 1:1 ratio. The prepared solution was left for a minimum of 16 h in a place inaccessible to light, at 4 °C. The solution was then diluted so that the absorbance of the cation radical solution measured at λ = 734 nm was in the range of 0.680–0.720. A Trolox standard curve was created using working standard solutions at concentrations ranging from 0 to 1125 μmol/L. Antioxidant capacity was given as μmol Trolox equivalent (TE) per 100 g of oil.

#### 3.2.9. Determination of Antioxidant Activity Using DPPH Cation Radicals

Antioxidant activity was also measured using the method described by Brand-Williams et al. [83] with modifications. For this purpose, a 0.6 mM solution of DPPH (1,1-diphenyl-2-picrylhydrazyl) was prepared and left for about 16 h in a dark place at 4 °C. The solution was diluted so that the absorbance measured at λ = 515 nm was in the range of 0.680–0.720. Then, 100 µL of the sample was measured into test tubes, and 4 mL of DPPH radical solution was added. The contents of the tubes were mixed using a Vortex centrifuge, and after 30 min of incubation in the dark, the absorbance against methanol as a blank was measured at λ = 515 nm. A Trolox standard curve was created as for ABTS antioxidant capacity assay. The result was given as μmol Trolox equivalent (TE) per 100 g of oil.

#### 3.2.10. Statistical Analysis

Statistical software Statistica 13 was used to statistically process the results obtained. One-factor analysis of variance (ANOVA) and Tukey’s post hoc test at the significance level of α = 0.05 were used for the following determinations: bioactive properties, quality parameters of oils, fatty acid profile of oils, and yield, while two-factor ANOVA was used for the results on the oxidative stability of the oils obtained. Microsoft Excel 2010 was used to calculate the mean and standard deviation. All analyses were carried out in triplicate for each sample tested. 

## 4. Conclusions

The results of this study demonstrate that the method of extraction significantly affects the yield, oxidative stability, quality parameters, and bioactive compounds content of PSO. UAE proved to be the most efficient technique in terms of oil yield, achieving 12.67% while maintaining favorable oxidative stability and moderate levels of bioactive compounds. Cold pressing, although less efficient in terms of yield, preserved the highest concentration of polyphenols and exhibited the strongest antioxidant activity, but showed the lowest oxidative stability and the highest susceptibility to lipid oxidation. Soxhlet extraction produced oil with the highest content of punicic acid but with exceeded recommended acid value limits, indicating possible hydrolytic degradation during extraction. The fitted statistical models, based on the Box–Behnken design, effectively predicted the extraction yield and oxidation parameters, confirming the reliability of the optimization process. Compared to conventional methods, UAE presents a promising alternative, balancing high efficiency with minimal degradation of thermolabile compounds and reduced environmental impact, aligning with circular economy and sustainability principles.

Future research should focus on quantifying individual bioactive compounds that may significantly influence the kinetics of lipid oxidation by altering reaction rates, activation energy, and even the underlying mechanism, depending on their concentration and stability. While some act as antioxidants, slowing the reaction, others may exhibit pro-oxidant effects under certain conditions. These findings highlight the need for more comprehensive studies to fully understand their role in the oxidative stability of pomegranate seed oil.

## Figures and Tables

**Table 1:** Results of ANOVA for Box–Behnken design model and equation fitting for ultrasound-assisted pomegranate seed oil extraction.

Response	Model Fitted	R^2^	CV (%)	Model *p*-Value	Lack of Fit *p*-Value
Yield	Linear	0.7544	5.45	0.0011	0.7319
τ_max_	Quadratic	0.9201	4.45	0.0274	0.1146

where τ_max_—maximum oxidation time, R^2^—determination coefficient, CV—coefficient of variation.

**Table 2:** Pomegranate seed oil extraction yield dependence on the applied method.

Oil	Yield [%]
CP_PSO	9.61 ^a^ ± 0.22
SE_PSO	11.49 ^b^ ± 0.19
UAE_PSO	12.67 ^c^ ± 0.48

CP_PSO—cold-pressed pomegranate seed oil; SE_PSO—pomegranate seed oil extracted by the Soxhlet method; UAE_PSO—pomegranate seed oil extracted by ultrasound-assisted extraction. The different lower-case letters indicate significantly different values (*p* ≤ 0.05). Data are presented as mean values followed by standard deviation (±SD).

**Table 3:** Fatty acid profile [%] of pomegranate seed oils.

	CP_PSO	SE_PSO	UAE_PSO
C16:0	2.45 ^b^ ± 0.06	2.27 ^a^ ± 0.02	2.42 ^b^ ± 0.02
C18:0	2.05 ^b^ ± 0.03	1.90 ^a^ ± 0.02	1.99 ^ab^ ± 0.04
C18:1 n-9c	4.96 ^c^ ± 0.08	4.54 ^a^ ± 0.04	4.75 ^b^ ± 0.06
C18:2 n-6c	4.72 ^b^ ± 0.08	4.42 ^a^ ± 0.04	4.51 ^a^ ± 0.05
C20:0	0.54 ^a^ ± 0.02	0.51 ^a^ ± 0.01	0.49 ^a^ ± 0.03
C20:1 n-9c	0.73 ^a^ ± 0.01	0.75 ^a^ ± 0.01	0.71 ^a^ ± 0.01
C18:3 (9c, 11t, 13c)	80.80 ^b^ ± 0.33	79.15 ^a^ ± 0.08	78.68 ^a^ ± 0.21
isomers of C18:3 (9c, 11t, 13c)	3.48 ^a^ ± 0.07	6.23 ^b^ ± 0.17	6.21 ^b^ ± 0.17
other	0.28 ^a^ ± 0.01	0.26 ^a^ ± 0.01	0.27 ^a^ ± 0.13
Σ SFA	5.04 ^c^	4.67 ^a^	4.89 ^b^
Σ MUFA	5.69 ^c^	5.28 ^a^	5.46 ^b^
Σ PUFA	86.56 ^b^	89.80 ^c^	84.88 ^a^

CP_PSO—cold-pressed pomegranate seed oil; SE_PSO—pomegranate seed oil extracted by the Soxhlet method; UAE_PSO—pomegranate seed oil extracted by ultrasound-assisted extraction SFA—saturated fatty acids; MUFA—monounsaturated fatty acids; PUFA—polyunsaturated fatty acids; The different lower-case letters show a statistically significant difference in the row at a *p* ≤ 0.05. Data are presented as mean values followed by standard deviation (±SD).

**Table 4:** Quality parameters of pomegranate seed oils.

Oil	AV [mg KOH/g]	PV [meq O_2_/kg]	p-AnV	TOTOX (2PV + p-AnV)
CP_PSO	2.23 ^a^ ± 0.10	4.68 ^b^ ± 0.15	15.78 ^b^ ± 0.21	25.14 ^c^ ± 0.21
SE_PSO	4.75 ^c^ ± 0.16	3.20 ^a^ ± 0.24	14.76 ^a^ ± 0.14	21.17 ^a^ ± 0.26
UAE_PSO	3.08 ^b^ ± 0.22	4.52 ^b^ ± 0.21	14.91 ^a^ ± 0.28	23.95 ^b^ ± 0.34

CP_PSO—cold-pressed pomegranate seed oil; SE_PSO—pomegranate seed oil extracted by the Soxhlet method; UAE_PSO—pomegranate seed oil extracted by ultrasound-assisted extraction; AV—acid value; PV—peroxide value; p-AnV—anisidine value. The different lower-case letters indicate significantly different values (*p* ≤ 0.05). Data are presented as mean values followed by standard deviation (±SD).

**Table 5:** Oxidation induction time (OIT) and oxidation time corresponding to the maximum intensity of oxidative changes (τ**_max_**) in pomegranate seed oils. Data were obtained at different temperatures.

Oil	90 °C		100 °C		110 °C		120 °C	
	OIT [min]	τ_max_ [min]	OIT [min]	τ_max_ [min]	OIT [min]	τ_max_ [min]	OIT [min]	τ_max_ [min]
CP_PSO	59.89 ^aD^ ± 0.76	61.72 ^aD^ ± 0.90	20.28 ^aC^ ± 0.08	20.96 ^aC^ ± 0.01	7.46 ^aB^ ± 0.04	7.81 ^aB^ ± 0.03	3.37 ^aA^ ± 0.26	3.75 ^aA^ ± 0.03
SE_PSO	104.60 ^bD^ ± 1.03	105.96 ^bD^ ± 0.83	39.13 ^bC^ ± 0.30	39.91 ^bC^ ± 0.30	14.57 ^bB^ ± 0.07	15.06 ^bB^ ± 0.10	4.67 ^bA^ ± 0.17	5.28 ^bA^ ± 0.27
UAE_PSO	105.11 ^bD^ ± 0.33	106.80 ^bD^ ± 0.64	40.00 ^bC^ ± 0.42	40.75 ^bC^ ± 0.39	14.66 ^bB^ ± 0.59	15.25 ^bB^ ± 0.40	5.18 ^bA^ ± 0.07	5.44 ^bA^ ± 0.02

CP_PSO—cold-pressed pomegranate seed oil; SE_PSO—pomegranate seed oil extracted by the Soxhlet method; UAE_PSO—pomegranate seed oil extracted by ultrasound-assisted extraction. The different lower-case letters indicate significantly different values (*p* ≤ 0.05). Data are presented as mean values followed by standard deviation (±SD). Different lower-case letters (a,b) in the columns indicate statistically significant differences between different samples oxidized at the same temperature but for different obtaining methods, at the level of α = 0.05. Different capital letters (A–D) in the rows indicate statistically significant differences between different samples obtained by the same method but at different temperatures, at the level of α = 0.05 (separately for OIT and τ_max_).

**Table 6:** Regression analysis of the DSC data at different temperatures for pomegranate seed oils.

Parameter	CP_PSO	SE_PSO	UAE_PSO
−*a*	5.83 ± 0.10	5.96 ± 0.10	6.15 ± 0.10
*b*	14.29 ± 0.10	14.39 ± 0.10	14.89 ± 0.10
*R* ^2^	0.9963	0.9998	0.9986
*E_a_* (kJ/mol)	106.15 ± 0.10	108.59 ± 0.10	111.99 ± 0.10
*Z* (min^−1^)	3.14 × 10^12^	3.87 × 10^12^	3.14 × 10^12^
*k* at 90 °C (min^−1^)	0.0017	0.0009	0.0090
*k* at 100 °C (min^−1^)	0.0043	0.0024	0.0025
*k* at 110 °C (min^−1^)	0.0106	0.0061	0.0064
*k* at 120 °C (min^−1^)	0.0247	0.0145	0.0157

CP_PSO—cold-pressed pomegranate seed oil; SE_PSO—pomegranate seed oil extracted by the Soxhlet method; UAE_PSO—pomegranate seed oil extracted by ultrasound-assisted extraction. *E_a_*—activation energy, *Z*—pre-exponential factor, *k*—oxidation reaction rate constant.

**Table 7:** Total polyphenol content (TPC), ABTS, and DPPH radical scavenging capacity of obtained pomegranate seed oils.

Oil	TPC [mg GAE/1 g of Oil]	Antioxidant Activity ABTS [µmol TE/100 g]	Antioxidant Activity DPPH [µmol TE/100 g]
CP_PSO	2.52 ^c^ ± 0.10	445.04 ^c^ ± 2.12	1666.19 ^c^ ± 6.33
SE_PSO	1.48 ^a^ ± 0.11	401.65 ^a^ ± 2.83	1482.94 ^a^ ± 4.88
UAE_PSO	2.08 ^b^ ± 0.14	418.69 ^b^ ± 3.29	1508.27 ^b^ ± 5.39

CP_PSO—cold-pressed pomegranate seed oil; SE_PSO—pomegranate seed oil extracted by the Soxhlet method; UAE_PSO—pomegranate seed oil extracted by ultrasound-assisted extraction. The different lower-case letters show a statistically significant difference in the column at a *p* ≤ 0.05. Data are presented as mean values followed by standard deviation (±SD).

**Table 8:** The Box–Behnken design variables for ultrasound-assisted extraction of pomegranate seed oil.

Run	X_1_-Amplitude (%)	X_2_-Time (minute)	X_3_-L/S Ratio
1	30	6	15
2	30	12	15
3	90	6	15
4	90	12	15
5	60	6	10
6	60	12	10
7	60	6	20
8	60	12	20
9	30	9	10
10	90	9	10
11	30	9	20
12	90	9	20
13	60	9	15
14	60	9	15
15	60	9	15

## Data Availability

Data are contained within the article.

## Abbreviations

The following abbreviations are used in this manuscript:

PSO	Pomegranate seed oil
PA	Punicic acid
FA	Fatty acid
CP_PSO	Cold-pressed pomegranate seed oil
SE_PSO	Pomegranate seed oil extracted by the Soxhlet method
UAE_PSO	Pomegranate seed oil extracted by the ultrasound-assisted method
UAE	Ultrasound-assisted extraction
GC	Gas chromatography
PDSC	Pressure differential scanning calorimetry
FAME	Fatty acid methyl esters
OIT	Oxidation induction time
L/S	Liquid to solid
TAG	Triacylglycerol
PUFA	Polyunsaturated fatty acid
MUFA	Monounsaturated fatty acid
SFA	Saturated fatty acid
AV	Acid value
PV	Peroxide value
p—AnV	Anisidine value
TPC	Total polyphenol content
CLnA	Conjugated linolenic acids
SHHE	Superheated hexane extraction
